# Beryllium Exposure Data: Rosenman et al. Respond

**Published:** 2006-04

**Authors:** Kenneth Rosenman, Mary Jo Reilly, Vicki Hertzberg, Carol Rice, Milton Rossman

**Affiliations:** Michigan State University, East Lansing, Michigan, E-mail: Rosenman@msu.edu; Emory University, Atlanta, Georgia; University of Cincinnati, Cincinnati, Ohio; University of Pennsylvania, Philadelphia, Pennsylvania

We thank Kolanz for his careful reading of our article (Rosenman et al. 2005). An erratum correcting the problem he noted appears on page A214.

How do these corrected numbers change our results? More chronic beryllium disease (CBD) and sensitization occurred with exposure below the Occupational Safety and Health Administration (OSHA) permissible exposure limit (PEL) of 2 μg/m^3^ (OSHA 2005) than we previously reported, but no CBD or sensitization was found below the Department of Energy (DOE) guidelines of 0.2 μg/m^3^ (DOE 1999) or the even more protective level proposed by the American Conference of Governmental Industrial Hygienists (ACGIH 2005) of 0.02 μg/m^3^. There was an insufficient number of individuals—only three, and they were all normal—to assess the safety of the DOE guidelines or proposed ACGIH level. Our previously reported findings regarding chemical and physical form—that sensitized individuals compared with individuals with CBD had higher exposure to beryllium in a soluble form and to fumes of beryllium (Rosenman et al. 2005)—remain unchanged. However, the mean peak exposure for the sensitized group in our Table 8, the mean nonsoluble exposure for the sensitized group in Table 9, and the mean dust exposure for the sensitized group and mean fume exposure for the CBD group in Table 10 were no longer significantly different, whereas the peak fume exposure for the CBD group was now significantly different in Table 10.

Having acknowledged and corrected the error pointed out by Kolanz, we take strong exception to his statement that “the study cannot sustain conclusions about the degree of risk associated with lower levels of exposure,” by which he means less than the OSHA PEL of 2 μg/m^3^ (OSHA 2005).

One of Kolanz’s criticisms of our article (Rosenman et al. 2005) is that the daily weighted average (DWA) represented the daily exposure based on data averaged over 3 months. Breslin and Harris (1958) conducted a time study, which was updated as activities and location changed for each job title. During a 3-month period, three or more samples were collected and standardized to represent the general area where each person worked, and in some cases the breathing zone during work activities. The arithmetic average of the samples for each location/type was then calculated and weighted by time (Breslin and Harris 1958). Use of these summed weighted values, divided by the shift duration, is consistent with the American Industrial Hygiene Association (AIHA) exposure assessment guidance (Mulhausen and Damiano 1998) cited by Kolanz.

We agree with Kolanz’s comment about the use of the flame spectroscopy method of chemical analysis and its general limit of detection of 0.1 μg/m^3^. In the job exposure matrix and task exposure matrix developed to support this project (Chen 2001), no exposure estimate was < 0.1 μg/m^3^, so this issue would have no effect on our results.

Kolanz also takes exception with our estimates of mean exposures, stating that these values were derived by triple averaging. We derived the mean exposures as follows: for a given worker in a given job in a given year, we multiplied the number of days worked in that year for that worker by our best estimated DWA for that job in that year. We then summed those values over all jobs for that worker to derive that worker’s cumulative exposure. Next we divided that worker’s cumulative exposure by the total number of days worked in his/her job history to derive that worker’s mean exposure. Thus, there is only one averaging on each worker and a subsequent averaging for the population. We used multiple metrics (cumulative, average, peak job, and peak task for total exposure; exposure by chemical form; and exposure by physical form) to characterize not only the central tendencies of the exposures but also their extreme excursions.

We stand by our statement that

The inclusion of genetic data combined with exposure data may better define which individuals in this cohort are at a particularly high risk of development of CBD and/or sensitization and may account for the absence of typical exposure–response seen with other environmental or occupational toxins.

It is also important to note that both peak exposure and the different chemical and physical forms may be important factors in the risk of development of CBD but are not part of the current OSHA standard (OSHA 2005).

Finally, Kolanz states that “Rosenman et al. (2005) made no recommendations regarding how to protect beryllium workers.” Clearly, however, our call to lower the allowable standard is a recommendation we put forth to protect beryllium workers. Despite the limitations of deriving historical exposure estimates, our corrected data continue to point to the inadequacy of the current OSHA standard to protect workers from developing chronic beryllium disease.
